# En-face analysis of short posterior ciliary arteries crossing the sclera to choroid using wide-field swept-source optical coherence tomography

**DOI:** 10.1038/s41598-021-88205-8

**Published:** 2021-04-22

**Authors:** R. Lejoyeux, R. Atia, K. K. Vupparaboina, M. N. Ibrahim, S. Suthaharan, J. A. Sahel, K. K. Dansingani, J. Chhablani

**Affiliations:** 1 Rothschild Foundation Hospital, 29 rue Manin, 75019 Paris, France; 2 XV-XX Hospital, 28 rue de Charenton, 75012 Paris, France; 3 Department of Ophthalmology, School of Medicine, University of Pittsburgh, 4200 Fifth Ave, Pittsburgh, PA USA; 4 Department of Electrical Engineering, Indian Institute of Technology Hyderabad, Telangana, 502285 India; 5 UPMC Eye Center, University of Pittsburgh, 203 Lothrop Street, Pittsburgh, PA 15213 USA; 6 Department of Computer Science, University of North Carolina ast Greensboro, Greensboro, NC USA

**Keywords:** Anatomy, Medical research, Optics and photonics

## Abstract

To study the topographic distribution of the short posterior ciliary arteries (SPCA) entry sites into the choroid in normal eyes using structural en-face swept source optical coherence tomography (SS-OCT). Retrospective analysis of SS-OCT scans (wide-field structural SS-OCT 12 × 12 mm) of 13 healthy subjects was performed. Cross-sectional swept-source OCT scans derived from a volume scan were represented as en-face image display following the Choroid-Scleral Interface to obtain en-face OCT. SPCAs in their last scleral location before choroidal entrance were identified manually, counted and localized by two masked observers. Correlations between two masked observers were analyzed using inter- and intra-class correlation. Accuracy for the choroidal inner and outer border segmentation was 95–99%. Eighteen eyes from 13 normal subjects were included for SPCA analysis. The mean number of arteries was 13.8 ± 3.5 per eye. Thirty-six percent were in the center of the posterior pole image; however, 21% were in the temporal part of the posterior pole. Median accuracy of the detection is 0.94. The correlation between the two observers was fair (0.54). Our algorithm allows visualization of the SPCA at the posterior pole of the eye using wide-field en-face SS-OCT. It can also help the clinicians to study the SPCAs in numerous ocular diseases, particularly its relationship with focal choroidal diseases.

## Introduction

Choroid is perfused by several posterior ciliary arteries (PCA) that originate from the ophthalmic arteries and traverse the sclera^1^. Posterior ciliary arteries arise from each part of the ophthalmic artery (first segment, second segment, or angle) and course towards the medial, temporal and superior aspects of the sclera before dividing into several arteries^2^.

Recently, with advancing imaging, choroidal vasculature is more and more implicated in the understanding and pathogenesis of retinal diseases^3–5^. Considering PCAs supplying choroidal vessels, it may be worthwhile to understand vessel entry into the choroid. With the advent of swept-source optical coherence tomography, there is a growing interest in the detection and visualization of short PCAs (SPCAs) in the posterior pole in-vivo^6^. There have been previous reports showing perforating vessels using OCT in myopes^7,8^. However, previous reports analyzed spectral domain OCT over a smaller area^9^.

Swept source OCT (SS-OCT) using a longer wavelength enabled a better visualization of the outer choroid, until the sclera. In some patients the PCAs are visible with structural SS-OCT^10^. Only the SPCAs coming from the lateral PCA are studied since the medial PCA is too far away from the center to be imaged. SPCAs are shown on cross sectional scans, however, topographic analysis in the entire posterior pole of healthy subjects of these vessels have not been done. In histologic section of sclera, there are between 10 and 20 SPCAs per eye followed by almost the same number of posterior ciliary nerves^11^. The usual variability in the number of SPCAs depends on the number of subdivisions of the PCA before it reaches the sclera region^12^. Current challenges include poor visibility of these vessels due to poor resolution and artefacts due to slicing at the chorio-scleral level.

Our aim is to establish an algorithm to visualize SPCAs entry sites and perform a topographic-based quantitative analysis of the SPCAs at the posterior pole using an en-face SS-OCT of the choroid-sclera interface.

## Results

Performance of automated choroid segmentation algorithm: As alluded earlier, 100 B-scans each from five dataset are taken to compare the algorithmic segmentation with manual segmentations performed twice by same observer.

### Accuracy of choroid inner boundary (CIB) detection

We observed that the overall mean dice coefficient (DC) obtained between proposed segmentation and the manual reference is 99.76% (0.16%) while that obtained between two manual segmentations is 99.79% (0.13%) indicating the efficacy of the proposed method. Completely details are presented in Table 1.

**Table 1:** Evaluation of automated CIB detection; Notation: *M1* manual segmentation-1, *M2* manual segmentation-2, *M* average of M1 and M2, *SD* standard deviation, *H1 to H5* represents datasets 1 to 5 (100 B-scans per dataset).

Dataset	Comparison	H1 Mean (SD) % (%)	H2 Mean (SD) % (%)	H3 Mean (SD) % (%)	H4 Mean (SD) % (%)	H5 Mean (SD) % (%)	Overall Mean (SD) % (%)
Healthy	Proposed vs M	99.76 (0.11)	99.84 (0.34)	99.65 (0.12)	99.77 (0.11)	99.78 (0.12)	99.76 (0.16)
	M1 vs M2	99.79 (0.12)	99.86 (0.33)	99.79 (0.07)	99.79 (0.05)	99.76 (0.07)	99.79 (0.13)

### Accuracy of choroid outer boundary (COB) detection

We observed that the overall mean DC obtained between proposed segmentation and the manual reference is 96.19% (1.28%) while that obtained between two manual segmentations is 96.99% (1.32%) indicating the efficacy of the proposed method (Table 2).

**Table 2:** Evaluation of automated COB detection; Notation: *M1* manual segmentation-1, *M2* manual segmentation-2, *M* average of M1 and M2, *SD* standard deviation, *H-1 to H-5* represents datasets 1 to 5 (100 B-scans per dataset).

Comparison	H1 Mean (SD) % (%)	H2 Mean (SD) % (%)	H3 Mean (SD) % (%)	H4 Mean (SD) % (%)	H5 Mean (SD) % (%)	Overall Mean (SD) % (%)
Proposed vs M	96.87 (1.16)	96.38 (1.22)	93.67 (2.04)	96.12 (0.95)	96.18 (1.05)	96.19 (1.28)
M1 vs M2	96.8 (1.35)	97.06 (1.25)	95.84 (1.75)	97.08 (1.70)	98.22 (0.53)	96.99 (1.32)

Overall, the proposed algorithm is observed to be accurate, consistent and performs at par with the manual delineation of the choroid.

### Subjects characteristics

Thirteen healthy subjects were imaged. Eighteen eyes of 13 subjects were included in the study. Eight eyes of 13 subjects could not be analyzed due to poor quality of the images. There were seven left eyes and 11 right eyes in the study. Best corrected visual acuity (BCVA) of every subject was 20/20 with mean spherical equivalent was − 0.77(SD = 2.7) diopters. Mean spherical equivalent of excluded eyes was not significantly different (− 2.5 (SD = 4.1) diopters).

#### Location of vessels

**Figure 1:**
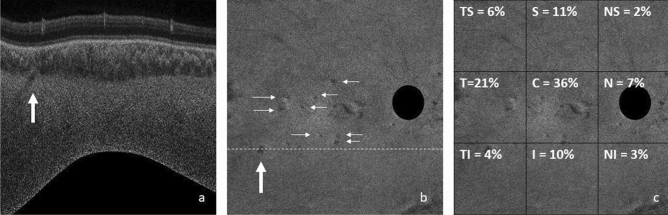
(**a**) Cross sectional B scan flattened around the COI that shows entry of short posterior ciliary artery (SPCA) (arrow) (1:1 pixel); (**b**) En-face scan obtained at the chorio-scleral junction showing entry sites of SPCAs. Dark-black area is the optic nerve location (1:1 pixel); (**c**) distribution of SPCAs over the posterior pole. Most common location is the center square (each square = 4 × 4 mm^2^) with a 36% entry site. Second most common is central temporal square (21%) (1:1 pixel).

The mean number of arteries localized in the patients were 13.8 (SD = 3.5). The distribution of number of arteries at the posterior pole is shown in Fig. 1.

Total 36% of the arteries were localized at the center of the posterior pole, 7% in the nasal part, 21% in temporal, 10% in the superior square, 11% in the inferior one. Overall, highest number of vessels (more than a third of the arteries) were present in the center of the posterior pole. However, 21% were in the temporal part of the posterior pole. Among the analyzed scans, there were no areas of the scan where the visualization was better which could bias the results.

#### Correlations

##### Autocorrelation

**Figure 2:**
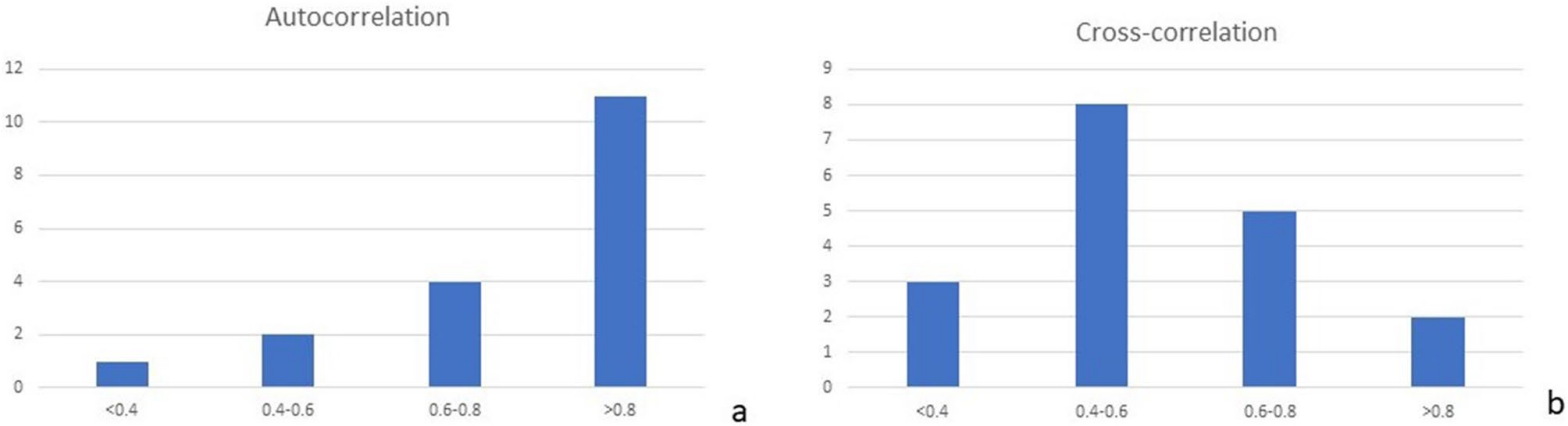
(**a**) Histogram showing the distribution of the Pearson correlation of the measures from a single observer (autocorrelations). (**b**) Histogram showing the distribution of the Pearson correlation between the masked observers (cross correlations).

In a histogram of the Pearson correlations of all 18 images, the distribution of the Pearson correlation is nonsymmetrical; hence, the statistical median is the measure of preference to quantify the central tendency of the correlation. Fifteen eyes had more than moderate correlation. (Fig. 2a). Thus, the median that we obtained for the 18 Pearson correlations that we computed is 0.9406 (very strong correlation).

##### Cross correlations

The cross correlation for each image between two masked observers showed moderate to high correlation in 15 cases (Fig. 2b) with the median cross correlation of approximately 0.54.

We also sorted the intra-class correlations and averaged the 9th and 10th correlations to obtain the median correlation measure; hence, the median accuracy of the detection is calculated 0.94.

## Discussion

Visualizing SPCA using in-vivo could be useful to understand chorioretinal and optic nerve diseases. We report visualization of SPCA branches entering into the choroid using SS-OCT. We localized the arteries with fair reproducibility and repeatability between masked observers. The mean number of arteries in the 12 × 12 mm scans is 13.8 ± 3.5. They are mainly located in the center of the posterior pole.

In histologic section of sclera, there are 10 to 20 SPCAs, depending on the number of divisions before the entrance in the sclera^12–14^. This number only takes into account the SPCAs emerging from the lateral PCA and not the medial one. There are also paraoptic PCAs that supply the optic nerve heads. Those are maybe the one identified in the nasal square of the OCT^15^.

Motaghiannezam et al. succeeded in imaging the very central SPCA vessels on cross sectional scans. However, distribution of vessels and quantification is not possible using cross sectional scans. Therefore, we used wide-field en-face OCT images and flattened the choroid-sclera interface to visualize, possibly, all entry sites of SPCA vessels into the choroid^6^. Puyo et al. also demonstrated SPCAs with cross sectional SS-OCT at the posterior pole in a patient. They confirmed the location of PCAs on laser doppler holography (LDH)^16^.

In-vivo, intrascleral vessels could also be localized using ICG angiography in eyes with choroidal atrophy^10^. In 66.9% of the eyes, it was localized in the foveal area, which is more than in our cohort. However, they included highly myopic eyes which may influence the visibility of scleral vessels. In our study, we included emmetrope subjects (mean spherical equivalent was − 0.77 (SD = 2.7) diopters), in whom scleral vessel visibility may be challenging due to choroidal thickness which is thinner in myopic eyes^10^ Flattening the Choroid-Sclera Interface was essential to count the number of scleral arteries. It enabled us to have is in a single stack of en-face image, to scroll up and down to avoid the “false positive” of SPCA that are easier to avoid if the image is parallel to the COB, to avoid edge artifacts on en-face images which may compromise on SPCA visualization. Rothenbuehler et al. could image the perforating scleral vessels but in a very short field (4.5 mm centered in the macula) using EDI^9^. With SS-OCT, we could perform images with wilder fields and deeper visualization.

Our technique has enabled us to recognize the arteries and avoid many false positives that could be either signal from the choroid and non vascular hyporeflective spots in the sclera. To improve the accurate detection, we used 5 to 30 substacks to avoid projection artifacts and confirmed the presence of arteries in correlation with cross-sectional B scans. There is already growing interest in analyzing the chorioretinal diseases in relation to scleral vessels. In high myopia, Ishida et al.^8^ and Watanabe et al.^17^ showed CNV next to the location of the scleral vessels. Moriyama et al.^18^ showed displaced PCAs entrance in the choroid around the boundaries of the staphyloma. Pedinielli et al.^7^ showed PCAs in the border of focal ectasia. However, in high myopia, the sclera is thin so that superficial PCAs could be mistaken for SPCAs because of the high remodeling of the choroid, sclera, and scleral vessels^10^. Amini et al. reported that the flow was not decreased in optic neuritis associated with multiple sclerosis in humans^19^. In choroideremia, scleral pits are scleral ectasias associated with choroidal and retinal interruption. Harvey et al. recently could colocalize scleral pits with the pathway of the posterior ciliary arteries. It could be a sign of the involvement of the scleral arteries in choroideremia^20^.

Also, in optic nerve disorders, there is an interest in analyzing the SPCA. In ischemic optic neuropathy associated with giant arteritis, the SPCA are the precise location of the occlusion^21^. In dogs, not only the lumen of the arteries but also their number was decreased in glaucomatous eyes^22^.

There are many limitations in our study. Small sample size and poor-quality images in eight eyes is one of the major limitations. We expect that improvement in penetration and resolution will help to improve the image quality and visualize more vessels. Also, we expected to visualize SPCA on OCT-A, however, due to poor decorrelation signal, we could not visualize any SPCA on SS-OCTA. We evaluated this technique only in healthy eyes, however, there could be challenges such as increased choroidal thickness or shadowing in diseased eyes which deteriorate scleral vessel visualization. Also, the trajectory of the arteries in the sclera is either oblique or horizontal. Therefore, the precise location of the hyporeflective structure as the entrance of the artery into the choroid remains uncertain. At the end, our images mainly focus on the temporal part of the optic nerve since the scans are macula-centered. Therefore, we could not evaluate SPCA branches on nasal part of the retina.

In conclusion, the use of the proposed technique on SS-OCT images of the posterior pole could identify short posterior ciliary arteries vascularizing the choroid in healthy subjects. This technique/tool is unique, and will help advance our current research in understanding choroidal vessels in several eye diseases. It can also facilitate our research for the automatic detection of blood vessels with an automated quantitative analysis. Our future research will left/right correlation and using 3D reconstruction, to find the location pattern of SPCA and compare with diseased eyes, which will further expand our understanding of vascularization patterns with choroidal pathologies.

## Methods

This study was carried out according to the Declaration of Helsinki and approved by the University of Pittsburgh Institutional Review Board. Informed consent was obtained for each healthy volunteer who underwent swept source OCT imaging. Every subject underwent best-corrected visual acuity assessment, a complete ophthalmologic examination, and a wide-field SS-OCT 12 × 12 mm in the Plex Elite 9000 device (Carl Zeiss Meditec, Dublin, CA) with fovea as the center. Swept source OCT scans were exported as complete 8-bit volumes. Each OCT volume consists of 1024 B-scans each of resolution 1024 × 1536. We included healthy subjects. Every subject with diabetes, high blood pressure, or any systemic disease or systemic treatment was excluded. Every subject with a history of maculopathy or retinopathy was excluded as well as high myopic eyes.

### Image analysis

#### Estimation of choroidal inner (CIB) and outer (COB) boundary

**Figure 3:**
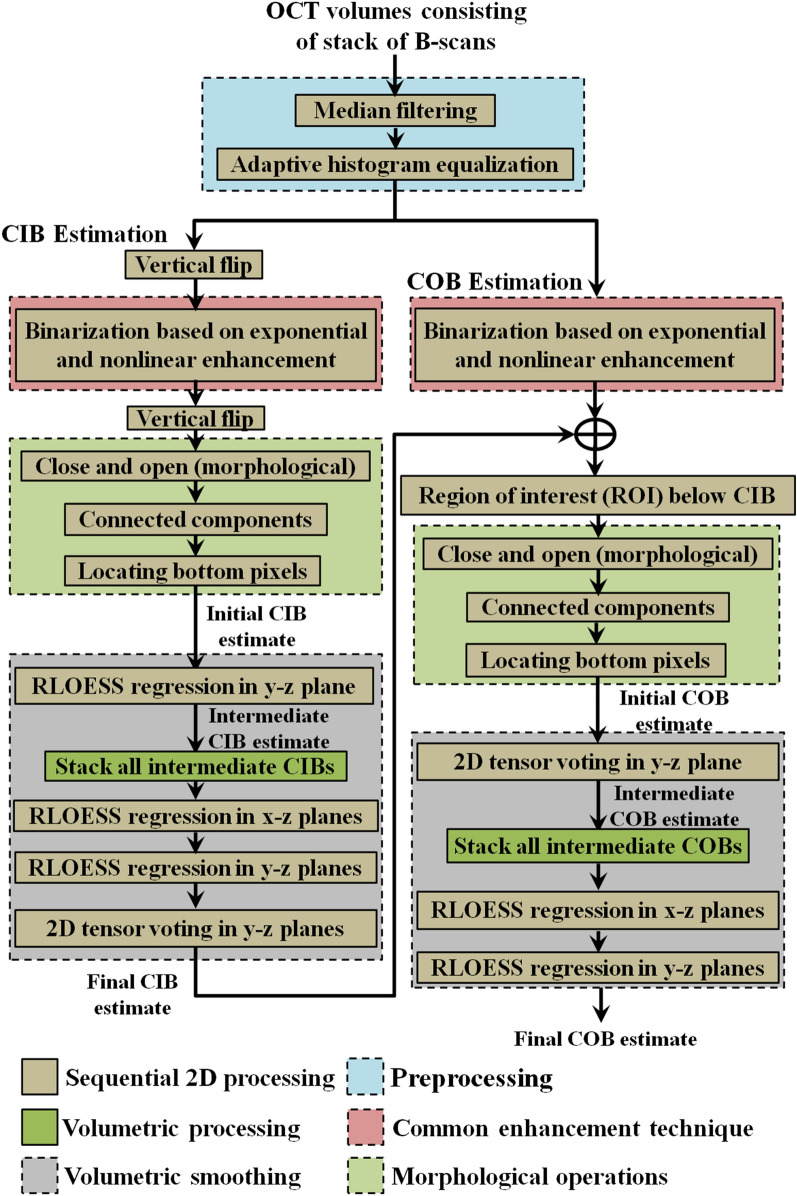
Schematic algorithm to demarcate the inner and outer choroidal boundaries (*CIB* choroidal inner boundary, *COB* choroidal outer boundary).

**Figure 4:**
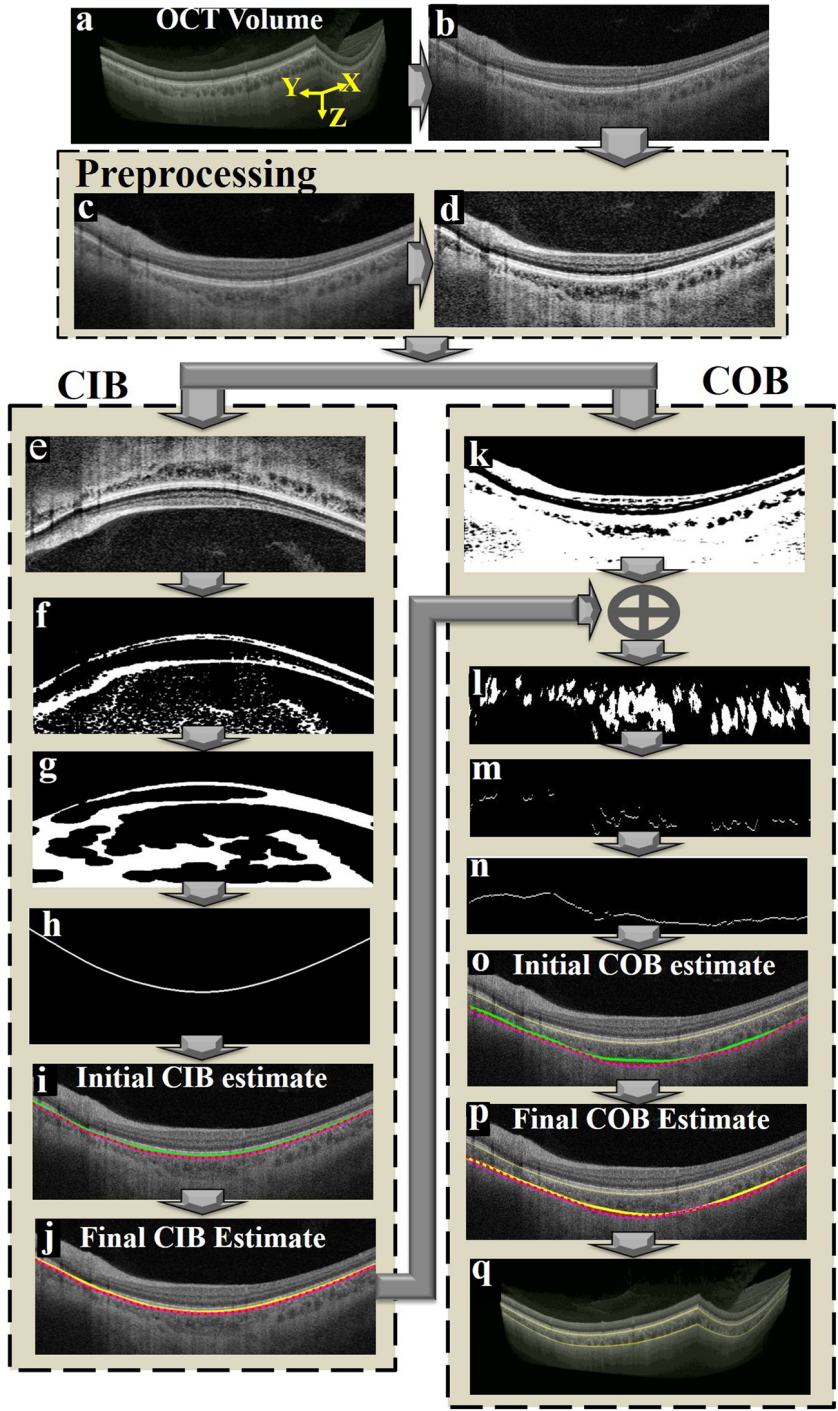
Graphical representation of the proposed algorithm; (**a**) a typical optical coherence tomography (OCT) volume scan; (**b**) a representative B-scan from the OCT volume to illustrate operations performed on a B-scan; (**c**) second order median filtered image; (**d**) adaptive histogram equalization; (**e**) vertically flipped image; (**f**) binarization based on two step exponentiation enhancement; (**g**) morphologically processed image; (**h**) detected CIB; (**i**) initial CIB estimate (green); (**j**) final CIB estimate (yellow); (**k**) binarization based on exponential and non-linear enhancement processes; (**l**) vessel section extracted image based on nal COB and morphological operations; (**m**) identification of extreme point in each column belonging to vessel sections towards sclera; (**n**) interpolation based on 2D tensor voting; (**o**) initial COB estimate (green) vs manual segmentations M1 and M2 (red and magenta); (**p**) final COB estimate (yellow) vs M1 and M2; and (**q**) Segmented boundaries on the whole volume (*CIB* choroidal inner boundary, *COB* choroidal outer boundary).

The structures in the OCT volume including sclera appear curved due to the spherical shape of the eye. Consequently, to get enface slices of the sclera it is required to restructure the OCT volumes to make the sclera flat. In other words, the scleral region below choroid-sclera interface (CSI) should be flattened. To this end, COB detection is a crucial step in localizing sclera below the choroid. We adopted a modified version of our previous reported method^23^ to segment COB. The previous method had two main limitations: (i) detection of CIB and COB was based on two separate approaches and the structural similarity (SSIM) technique employed earlier for obtaining initial COB estimate was computationally intensive; and (ii) individual B-scan processing was performed and inter-slice dependency within the B-scans of a OCT volume was not considered while detecting CIB and COB which may not result in seamless (spatially consistent) boundaries across B-scans. In view of this, in the modified methodology we made two new contributions to address the aforementioned limitations. In particular, they include (i) obtaining initial estimates of both CIB and COB based on single technique i.e., using a two-step exponentiation enhancement method inspired by idiosyncrasies of the OCT imaging; and (ii) performing volumetric smoothing, to correct spurious detection in initial estimates of CIB and COB, leveraging the inherent neighborhood dependency among the B-scans of an OCT volume. The schematic of the proposed method for detecting CIB and COB is depicted in Fig. 3, and corresponding implementation output of each step are shown in Fig. 4 based on a representative OCT B-scan (Fig. 4b) of an OCT volume (Fig. 4a). The coordinate orientation of the XYZ axis of OCT volume is also depicted in Fig. 3a. In particular, the proposed method involves (i) detecting initial CIB estimate sequentially in each B-scan and stack all estimates in 3D; (ii) obtaining final estimate of CIB by smoothing in orthogonal direction to the B-scan orientation; (iii) detecting initial COB estimate sequentially in each B-scan and stack all estimates in 3D; and (iv) obtaining final COB estimate by adopting step (ii) of CIB to COB. Step-by-step details of the method are described in the following.

##### Pre-processing

In the OCT image acquisition process, the attenuation of the signal happens more towards the deeper regions, resulting in poor contrast between the vessel region and stromal region in the choroidal layer. Further, there is inherent speckle noise due to coherence of light. To overcome the aforementioned limitations and improve the signal-to-noise ratio, we apply certain preprocessing steps. In particular, second order median filtering with 6 × 6 tiling and adaptive histogram equalization are applied on the B-scans to reduce the inherent speckle noise and enhance the contrast of the image, respectively (Fig. 4c,d). Further, the exponentiation enhancement employed in the subsequent steps compensates for the signal attenuation.

##### Common enhancement method

The core techniques employed to obtain initial estimates of CIB and COB are exponentiation and nonlinear enhancement. We employed these operations for binarizing OCT B-scan for choroidal stromal-luminal area quantification^24^. These operations are performed on raw OCT intensity scale. Accordingly, for a grayscale intensity B-scan image $I,$ raw intensity for each pixel $\left( {x,y} \right)$ is obtained by

1 $$I_{raw} \left( {x,y} \right) = \left( {\frac{{I\left( {x,y} \right)}}{255}} \right)^{4} .$$

The exponential enhanced image of $I_{raw}$ is obtained by ^25^.

2 $$I_{expenh} \left( {x,y} \right) = \left( {\frac{{I_{raw} \left( {x,y} \right)}}{{2\mathop \sum \nolimits_{k = x}^{p} I_{raw} \left( {k,y} \right)}}} \right)^{n} ,$$

where $n$ is the exponent and we empirically chose $n = 10$. In general, exponentiation enhancement compensates for the signal attenuation suffered at deeper posterior segment layers during OCT image acquisition.

Further, the nonlinear enhanced image of $I_{expenh}$ is given by

3 $$I_{nonlin} \left( {x,y} \right) = x^{2} I_{expenh} \left( {x,y} \right).$$

The aforementioned operations applied while estimating CIB is slightly different from those applied while estimating COB. In particular, for CIB detection they are applied on a vertically flipped B-scan to enhance retinal structures while for COB detection they are applied on original B-scan orientation to enhance choroidal structures. The details are described in the following.

##### Choroid inner boundary (CIB) detection

CIB detection is performed to localize the choroid region below retina to facilitate COB detection. As alluded earlier, we first obtain an initial CIB estimate for each B-scan and then smooth them across B-scans to obtain a final smoothed CIB estimate.

*Initial CIB estimate* We employed exponential and non-linear enhancement operations (Eqs. (1)–(3)) on a flipped OCT image (Fig. 4e) to enhance retinal substructures while attenuating the intensities corresponding to the choroid and the sclera (Fig. 4f). Specifically, intensities of the retinal pigment epithelium (RPE) and other retinal layers get saturated because of their higher row indices at the bottom of the image. This is an inverse operation described for estimating stromal-luminal ratio of the choroid reported earlier^24^. Subsequently, thresholding is performed using a mid-grayscale intensity threshold of 128 to obtain a binarized image. This results in closely placed large connected components of the retinal region. There could be some small spurious components as well. Accordingly, to spurious components and to detect only large connected components belonging to the retinal layers, morphological operations including close, open and connected components algorithm (Fig. 4g) is employed. Subsequently, to obtain the initial CIB estimate (Fig. 4h,i). Note that the aforementioned operations will be again employed in the detection of COB in the subsequent steps.

*Smoothing in the orthogonal direction* We noticed some spurious detections in the initial CIB estimates of some of the B-scans of the OCT volume especially when there is deformed or depleted retinal pigment epithelium (RPE). In view of this, we proposed to perform smoothing on the stack of all initial CIB estimates of the volume in the orthogonal direction ($x{\text{-}}z$ plane) to the plane containing B-scans. In particular, we want to leverage the neighborhood interdependency among the boundary estimates to correct the deviations. For each array of CIB estimates in the orthogonal direction, we applied robust locally estimated scatterplot smoothing (RLOESS)^26^. Following it, we applied tensor voting^23,27^ (adopted from our previous work^23^) in the original B-scan direction ($y{\text{-}}z$ plane) to smoothen out minor deformations representing the final CIB estimate (Fig. 4j).

##### Choroid outer boundary (COB) detection

COB detection also involves processing of B-scans in two stages including scan-wise detection of initial COB estimates and correction of the spurious detections if any in the volume by performing smoothing in the orthogonal direction to the plane containing B-scans.

*Initial COB detection* We primarily replaced the structural similarity (SSIM) index part in our earlier work with the exponentiation and nonlinear enhancement operations (Eqs. (1)–(3)). However, unlike during CIB detection, they are applied in the original orientation without flipping the B-scan (Fig. 4k,n). Subsequently, tensor voting^23,27^ is employed to generate a continuous smooth initial COB estimate (Fig. 4o).

*Smoothing in the orthogonal direction* Similar to CIB initial estimate, we noticed that the initial COB estimate deviates from the actual boundary in few B-scans. Hence, to surmount those outliers, we again performed RLOESS^26^ smoothing on the stack of initial COB estimates in the direction orthogonal to the plane containing OCT B-scans (Fig. 4p).

The detected CIB & COB for the whole OCT volume under consideration is depicted in Fig. 4q.

##### Scleral en-face image extraction

**Figure 5:**
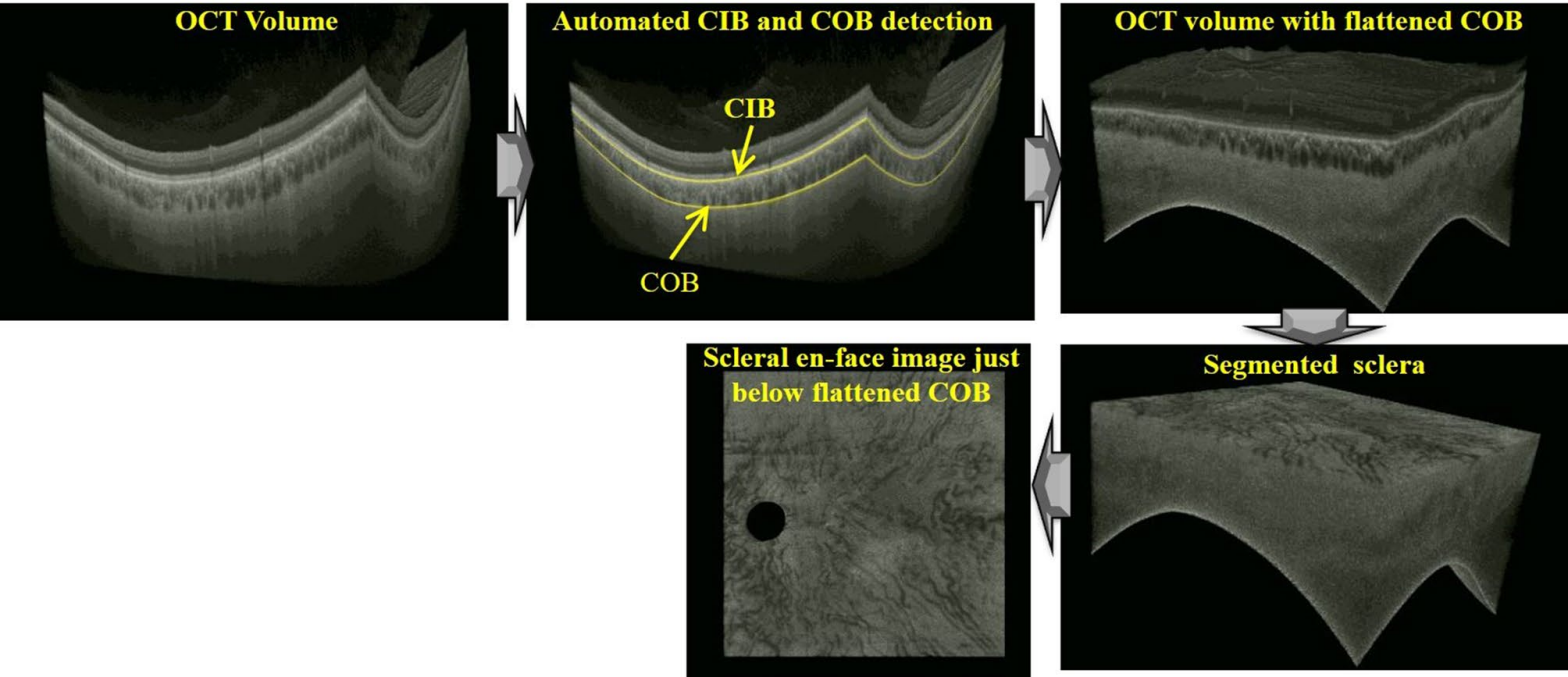
End-to-end depiction of automated extraction of volume below the sclera-choroid interface scans based on the representative wide-field optical coherence tomography (OCT) volume.

As alluded earlier, the structures in the OCT volume including sclera take spherical shape due the anatomical structure of the eye. Therefore, it is required to flatten COB (choroid-sclera interface) to obtain scleral enface slices. To this end, once the COBs for the entire OCT volume is obtained, the pixel coordinates of the OCT volume are transformed so that the COBs of the all B-scans lie on a plane. On the representative OCT volume, Fig. 5 depicts the OCT volume with flattened COB, the OCT volume with peeled off retinal and choroidal layers, and finally the extracted scleral en-face image*.*

#### Validation of choroid segmentation algorithm

Choroidal segmentation algorithm was validated for choroidal inner border (CIB) and choroidal outer border (COB) separately using 100 scans randomly chosen from each of the five datasets (total 500 scans). The performance of automated detection of CIB and COB was validated by considering the intraobserver variability as the reference. Manual delineations were performed twice by an expert using the ImageJ software. Delineations performed in the first round are masked to the second round of markings. In particular, first an initial piece-wise linear boundary was marked by picking sparse locations on the boundary using the `Segmented Line' tool. Subsequently, a smooth boundary was obtained by fitting a spline to the initial boundary by ‘Fit Spline’ operation (Edit → Selection → Fit Spline).

##### Dice coefficient (DC) to validate choroid segmentation

We adopted widely accepted Dice coefficient (DC) metric to compare accuracy algorithmic segmentation of CIB and COB vis-à-vis corresponding manual segmentations and is defined in the following.

Let $x_{ij}$ and $y_{ij}$ represents the two thickness measurements at i-th $\left( {i = 1, \ldots ,N} \right)$ A-scans2 of j-th B-scan. Further, the pixel indices corresponding to $x_{ij}$ and $y_{ij}$ are represented by $C_{xij}^{{}} \left( {\left| {C_{xij}^{{}} } \right| = x_{ij} } \right)$ and $C_{yij}^{{}} \left( {\left| {C_{yij}^{{}} } \right| = y_{ij} } \right)$. Then the Dice coecient (DC) at A-scan location $ij$ is defined by

4 $$DC_{ij} = \frac{{2\left| {C_{xij} \cap C_{yij} } \right|}}{{\left| {C_{xij} \left| + \right|C_{yij} } \right|}}.$$

The value of DC = 1 (100%) indicates that two measurements are equal and DC = 0 indicates that two measurements are completely uncorrelated.

We considered DC between the two manual segmentations as the yardstick and compared with DC between the proposed algorithmic segmentation and the reference manual segmentation obtained by averaging two manual segmentations.

##### Localizing the arteries

The OCT volumes were then rebuilt with A-scan positions adjusted in *z* to align the chorio-scleral junction to a plane. A substack of 5 to 30 en-face projections was chosen manually, to select the best stacks showing the arteries.

The observer marked manually the arteries which could be followed between the stacks and which entered the choroid. Nine-squared squares (each 4 × 4 mm^2^) were determined from the en-face image. The number of PCAs in each sector was determined using their location. Measurements of the vessels in this manner were performed twice by the two masked observers. Masked observers were masked to their first measurements as well as other observer’s measurements. Eyes with identification of less than 10 PCAs due to low contrast were secondarily excluded from the analysis because it is the minimum number of SPCAs encountered from histological studies^12^.

### Statistical analysis

Correlation analysis (e.g., Pearson correlation) has been used for determining choroidal vascular density^3–5^ and developing a feature learning technique^28^. In our approach, interclass (cross correlation) and intraclass correlations (autocorrelation) were determined to SPCA entry site detection. Statistical analysis was made using ExcelStats. Suppose X and Y are the pixel-based positional information of an artery on a 2D image plane, by considering the bottom left corner as the origin of the image pixels in a cartesian coordinate system. Then, we define Z as the positional information of the artery that was determined by a human expert such that

5 $$\left| {Z - \sqrt {X^{2} + Y^{2} } } \right| < \epsilon ,$$

where $\epsilon$ determines the robustness of the artery detection. We then used correlation between Z_t_ and Z_t+1_ as a measure of determining the artery positions on an image, where Z_t_ and Z_t+1_ satisfy the above equation at trial t with n images (we used n = 18).

Then the Pearson correlation is calculated in autocorrelation and cross correlation. Qualification of the correlation is based on the empirical characterization of Divaries correlation coefficient^29,30^, which defines the following groups:

- below 0.20 → very weak correlation;
- 0.20 to 0.40 → weak correlation;
- 0.40 to 0.60 → moderate correlation;
- 0.60 to 0.80 → strong correlation;
- above 0.80 very strong correlation.

Distribution of arteries (percentages) wsas analyzed in 9 sectors (each square = 4 × 4 mm^2^) over the posterior pole.

## Abbreviations

CIB: Choroidal inner boundary
COB: Choroidal outer boundary
CSI: Choroid-sclera interface
DC: Dice coecient
LDH: Laser doppler holography
RLOESS: Robust locally estimated scatterplot smoothing
RPE: Retinal pigment epithelium
SPCA: Short posterior ciliary arteries
SSIM: Structural similarity
SS OCT: Swept source optical coherence tomography
